# Body Context and Posture Affect Mental Imagery of Hands

**DOI:** 10.1371/journal.pone.0034382

**Published:** 2012-03-30

**Authors:** Silvio Ionta, David Perruchoud, Bogdan Draganski, Olaf Blanke

**Affiliations:** 1 Laboratory of Cognitive Neuroscience, Brain-Mind Institute, Ecole Polytechnique Fédérale de Lausanne, Switzerland; 2 LREN, Département des Neurosciences Cliniques, CHUV, Université de Lausanne, Switzerland; 3 Max Planck Institute for Human Cognitive and Brain Sciences, Leipzig, Germany; 4 Mind Brain Institute, Charité and Humboldt University, Berlin, Germany; 5 Department of Neurology, University Hospital, Geneva, Switzerland; McMaster University, Canada

## Abstract

Different visual stimuli have been shown to recruit different mental imagery strategies. However the role of specific visual stimuli properties related to body context and posture in mental imagery is still under debate. Aiming to dissociate the behavioural correlates of mental processing of visual stimuli characterized by different body context, in the present study we investigated whether the mental rotation of stimuli showing either hands as attached to a body (hands-on-body) or not (hands-only), would be based on different mechanisms. We further examined the effects of postural changes on the mental rotation of both stimuli. Thirty healthy volunteers verbally judged the laterality of rotated hands-only and hands-on-body stimuli presented from the dorsum- or the palm-view, while positioning their hands on their knees (front postural condition) or behind their back (back postural condition). Mental rotation of hands-only, but not of hands-on-body, was modulated by the stimulus view and orientation. Additionally, only the hands-only stimuli were mentally rotated at different speeds according to the postural conditions. This indicates that different stimulus-related mechanisms are recruited in mental rotation by changing the bodily context in which a particular body part is presented. The present data suggest that, with respect to hands-only, mental rotation of hands-on-body is less dependent on biomechanical constraints and proprioceptive input. We interpret our results as evidence for preferential processing of visual- rather than kinesthetic-based mechanisms during mental transformation of hands-on-body and hands-only, respectively.

## Introduction

Mental imagery is a cognitive task commonly used in daily life, during which, even in the absence of sensory stimulation, inner mental representations are activated and possibly determine an almost-perceptive experience [1]. A special class of mental imagery is mental rotation - the ability to mentally rotate representations of two- or three-dimensional objects (e.g. shapes, letters, numbers, etc.), or bodily stimuli (e.g. hands, faces, bodies, etc.) [review in 2], [3]. The investigation of mental rotation of body parts is classically performed by visual presentation of a body part followed by judgment of its laterality [4]. During such mental rotation participants tend to imagine their own body part as moving towards the stimulus [5]. The response times (RTs) required to align a rotated stimulus to the vertical follow a psychophysical profile that is progressively increasing RTs for stimuli presented from 0° to 180° and progressively decreasing RTs for stimuli oriented from 180° to 360° [6]. Moreover, mental rotation of body parts is sensitive to proprioceptive information, leading to longer RTs for judging the laterality of stimuli oriented in anatomically difficult positions [5], [7], [8]. In addition several studies showed that if participants keep their hands in more awkward postures during mental rotation of hands, their performance is slower with respect to when their hands are kept in more natural postures [e.g. 9]. This posture effect is highly specific because it is present only in the mental rotation of stimuli representing the body segment whose posture is manipulated and not for other body parts, e.g. for hands but not feet [10], [11]. The influence of biomechanical constraints and proprioceptive information relative to posture on mental rotation, leads to the idea of an embodied cognitive processing that is classically referred to as motor imagery [12]. The embodied nature of motor imagery is further supported by many studies that adopted different approaches such as neuroimaging [e.g. 13], transcranial magnetic stimulation [e.g. 14], clinical work [e.g. 15], [16] and empirical investigation [e.g. 17], and that consistently showed that mental rotation of body parts shares neural mechanisms with movement planning and execution.

Despite the consistent findings on the relationship between stimulus orientation and RTs in the mental rotation of body parts, several studies reported that mental rotation of stimuli representing whole-bodies is less dependent on stimulus orientation, that is the orientation-dependent profile of RTs is less pronounced or even absent for this class of stimuli [18], [19], [20], [21], [22], [23]. However, similarly to studies investigating mental rotation of hands, even in studies using whole-body stimuli participants are generally asked to judge the lateralization of a particular body part (usually one hand or one arm). In this view, the whole-body stimuli that have been tested in mental rotation studies can be seen as a variant of the hand stimuli, because they still contain only one arm/hand that is crucial for an accurate performance. For this reason hereafter we will define as “hands-only” those stimuli representing only one hand (without the rest of the body), and as “hands-on-body” those stimuli representing a marked hand attached to a body.

Up to now, most experimental and clinical work investigated either mental rotation of only hands-on-body stimuli [e.g. 22], [24], [25], [26], or only hands-only stimuli [e.g. 10], [11], [27], [28], [29]. This renders it difficult to directly compare the effects of different experimental manipulations on the processing of these two different stimuli. Most of the studies in which the same subjects were asked to perform mental rotation of two different stimuli used hands-on-body versus objects [e.g. 30], [31]. Several clinical studies investigated mental rotation of different classes of stimuli in the same patients, but compared hands-only to objects [32], [33], or to different body parts [34], [35]. In only two studies, the same healthy subjects [18] or patients [36] performed mental rotation of hands-only and hands-on-body. Yet, the comparison between these stimuli is also limited in these studies due to the fact that the target hand was different (or even absent) between the two classes of stimuli. To overcome limitations of the previous studies and in order to provide evidence on the potentially differential mechanisms involved in the mental rotation of hands-only versus hands-on-body, a better controlled direct comparison between the two stimuli is required. To this aim in the present study we asked the same participants to perform mental rotation of hands-only and hands-on-body stimuli. Importantly the target hands were presented in exactly the same orientation and view in both types of stimuli. This within-subject comparison of stimuli with homologues physical features has to the best of our knowledge not yet been investigated.

It has been recently suggested that in order to perform mental spatial transformations, it is possible to adopt at least two different strategies: during so-called “perspective” transformations the relationship between the coordinates of an object and of the environment is fixed but there are changes of the participant's point of view; during “effector-based” transformations there are changes in the effector-centered reference frame with respect to both the object-centered and the environmental frames [review in 37]. The selection of one or the other imagery strategy depends also on the stimulus: hands-only should elicit effector-based transformations and the activation of more somatosensory and kinesthetic representations; hands-on-body should elicit perspective transformations and more visuo-spatial representations [37]. However the specific direct comparison between these two stimuli is still missing.

Using more comparable target hands with respect to previous studies and adopting a within-subject design, our approach may elucidate potential differences that have been so far only suggested based on theoretical speculations. If the mental rotation of the hands-only relies on effector-based transformation and mental rotation of hands-on-body relies on perspective transformations, we expect that the former will be also more sensitive to stimulus orientation. Moreover if mental processing of hands-only evokes more somatosensory representations and hands-on-body activates visuo-spatial representations, we predict that the information brought by the stimulus view (e.g. dorsum or palm) would have a strong effect on the mental rotation of hands-only, and less or no effect on the mental rotation of hands-on-body.

Finally, while the influence of proprioceptive inputs (e.g. posture) on mental rotation of hands-only has been consistently reported, the corresponding data on mental rotation of hands-on-body stimuli is missing. Indeed, mental rotation of hands-on-body stimuli has been reported to be both dependent [30] and independent [31] of motor and proprioceptive representations. In order to disambiguate the potentially differential impact of proprioception on mental rotation of hands-only and hands-on-body stimuli, we manipulated the participants' posture while performing the tasks and hypothesized that posture should influence mental rotation of hands-only but not hands-on-body stimuli. Accordingly, for more awkward postures we expected longer RTs in the mental rotation of hands-only stimuli but not (or less pronounced) of hands-on-body stimuli.

## Materials and Methods

### Participants

Thirty healthy participants (8 females) aged 18–40 years (M = 23.3 years, SD = 4.6) were enrolled in the experiment after signing a written informed consent prior to the experiment. All participants were right-handed according to the revised Edinburgh Handedness Inventory (M = 97, SD = 8) [38]. The local Ethical Committee of the Ecole Polytechnique Fédérale de Lausanne (EPFL; Switzerland) approved the experiment, which was in accordance with the Declaration of Helsinki (1964). Participants gave written informed consent prior to inclusion in the study.

### Stimuli

**Figure 1:**
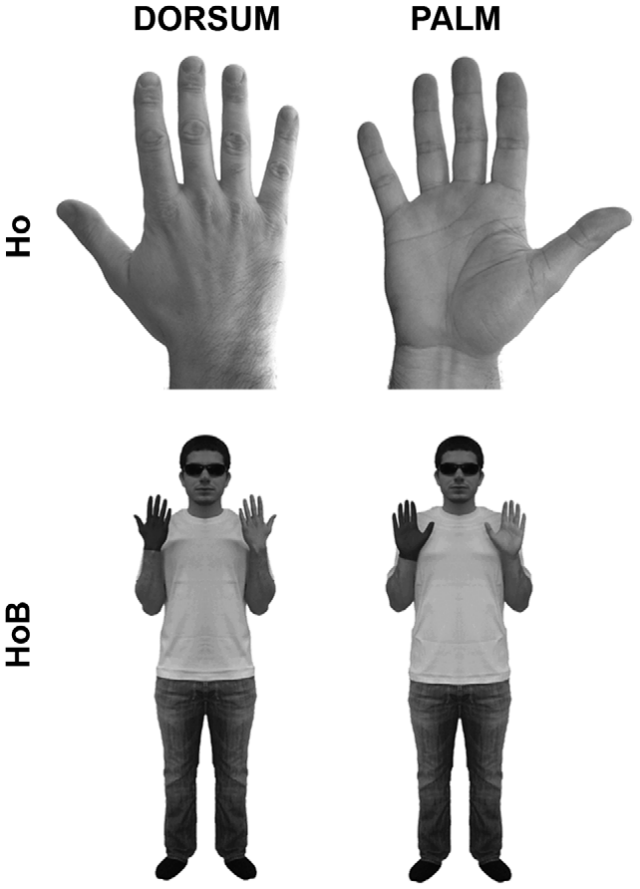
Experimental stimuli. The hands-only (hands-only) stimuli represented one hand from the dorsum- and the palm-view. The hands-on-body (hands-on-body) stimuli represented a human body with one hand darker than the other, shown from the dorsum- and the palm-view. All stimuli were rotated in four orientations. The overall configuration of the hands was very similar between the hands-only and hands-on-body stimuli.

The experimental hands-only stimuli consisted of naturalistic pictures of hands [Fig. 1; [10], [11]]. Left-lateralized hands-only stimuli were mirror images of the right-lateralized exemplars. Participants were presented one hand at a time. The hands-on-body stimuli were adapted from the original studies by Ratcliff [39] and Zacks and Tversky [40], and represented a realistic front-facing whole-body standing straight, with the elbows bended and the forearms upright such that the hands were level with the shoulders (Fig. 1). One hand of the hands-on-body stimuli was darker than the other. Left-lateralized hands-on-body stimuli were mirror images of the right-lateralized ones. Both the hands-only and the hands-on-body stimuli presented either the palm or the dorsum view of the hands. Importantly, between the dorsum and the palm view the overall configuration was very similar in both hands-only and hands-on-body. All stimuli were oriented in one of four clockwise orientations from the upright (0°, 90°, 180°, 270°). The upright orientation was defined as fingers pointing upwards (0°). All stimuli were presented one at a time on the computer screen, and covered a visual angle of 11.5°–13.7°.

### Procedure

The experimental session consisted of four blocks. Each block contained 48 stimuli of only one category (hands-only or hands-on-body). Stimuli varied in terms of type (hands-only, hands-on-body), laterality (left, right), view (dorsum, palm), and orientation (0°, 90°, 180°, 270°). Each stimulus was randomly presented 3 times in each block. The two blocks within each stimulus type varied in terms of hand posture. In one condition participants placed their hands on their knees (front condition), in the other hands were held behind their backs (back condition). In both the front and the back conditions, the hands were not visible to the participants [see 11 for more details]. The order of conditions and blocks were counterbalanced across participants.

Participants sat in front of a computer screen. Stimuli presentation was controlled with E-Prime2 (Psychology Software Tools Inc., Pittsburgh USA). At the beginning of each trial participants fixated a cross for 1000 ms. Then the stimulus appeared and participants were asked to verbally judge as quickly and accurately as possible its laterality. For the hands-only, participants were presented one hand and judged its laterality. For the hands-on-body, participants were asked to indicate the laterality of the darker hand. Each stimulus remained visible on the screen until the participant gave a response. RTs were recorded from a microphone positioned in front of the participant. Accuracy was manually recorded by the experimenter. Participants' gaze was continuously monitored by the experimenter. Before the beginning of the experimental session, participants were trained in the task using ten stimuli (five hands-only and five hands-on-body) oriented differently with respect to the ones of the real experiment, in order to avoid any practice bias. Posture was not manipulated during the training phase.

### Data Analysis

We analyzed only RTs, defined as the time between the onset of the stimulus and the participant's verbal response, based on previous studies showing that orientation and view particularly affect RTs [4], [5], [29], [41], [42]. Several previous studies showed that RTs required for mentally rotate body parts are comprised within 500 ms and 3500 ms [4], [5], [30], [41], [42], [43], [44], [45], [46]. For these reasons we excluded trials with incorrect responses and with RTs longer than 3500 ms or shorter than 500 ms from analysis, with a total loss of 8.6% of the trials. RTs were analyzed using a 5-way repeated measures ANOVA with stimuli (hands-only, hands-on-body), hand posture (front, back), stimulus laterality (right or left), view (palm, dorsum), and orientation (0°, 90°, 180°, 270°) as main factors. Post-hoc comparisons were carried out using Newman-Keuls test with a significance limit at p<0.05. For statistical analysis we used the STATISTICA software (StatSoft Inc., Tulsa, US).

## Results

### Stimulus-related effects

**Figure 2:**
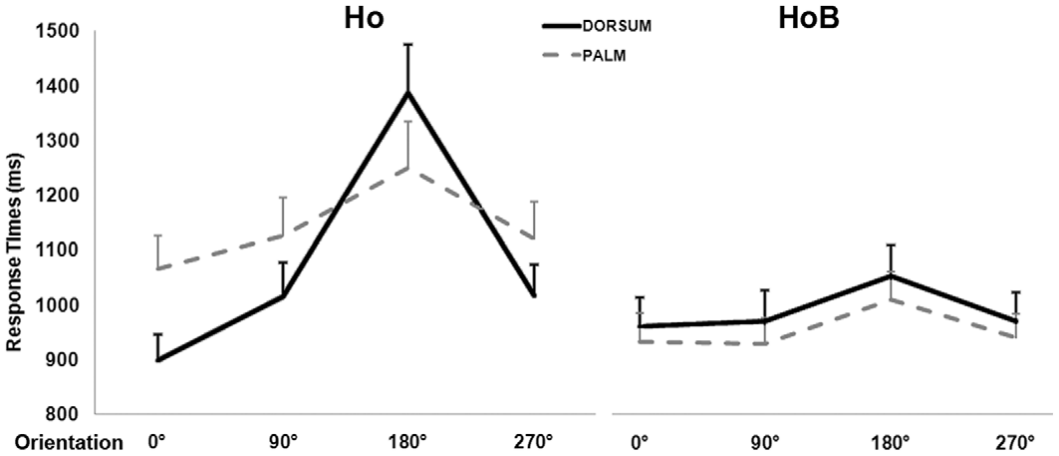
Mental rotation of hands-only and hands-on-body is differentially influenced by view and orientation. Mental rotation of hands-only (hands-only) is modulated by the stimulus view, with more orientation-dependent RTs for stimuli presented from the dorsum- with respect to the palm-view. Mental rotation of hands-on-body (hands-on-body) is less view- and orientation-dependent. Error bars represent standard errors.

There was a significant 2-way interaction between stimulus by orientation [F(3,78) = 12.4, p<0.01], showing that for the hands-only stimuli the typical profile of RTs for mental rotation was preserved, with the slowest responses for stimuli oriented at 180° (1338 ms) with respect to all the other orientations (all p<0.01). Conversely, the stimulus orientation-related differences of RTs were not significant for the hands-on-body, indicating that the distribution of RTs as a function of orientation was not preserved for mental rotation of hands-on-body stimuli. Additionally, in the significant 2-way interaction between stimulus and view [F(1,26) = 7.99, p<0.01], the difference between dorsum and palm views was significant for the hands-only (1095 ms and 1141, respectively; p<0.037), but not for the hands-on-body (1005 ms and 968 ms, respectively; p = 0.08). The 3-way interaction between stimulus, view and orientation [F(3,78) = 16.66, p<0.01] demonstrated that the RTs profile for the hands-only was more orientation-dependent for the stimuli showed from the dorsum view than from the palm view. This view-related difference of the RTs profiles was not significant for the hands-on-body stimuli (Fig. 2). The 3-way interaction between stimulus, laterality and orientation [F(3,78) = 5.42, p<0.01] showed that the mental rotation function is preserved for the hands-only, with the typical increase of RTs as a function of orientation for both right- and left-lateralized stimuli. Moreover, with hands-only stimuli presented at 270°, right-lateralized ones (992 ms) were judged faster (p<0.01) than left-lateralized ones (1163 ms). This was not the case for the hands-on-body stimuli, where the RTs were less modulated by orientation equally for the right- and left-lateralized, independent of stimulus angle. Finally, the statistical analysis of RTs showed a significant main effects of stimulus [F(1,26) = 12.96, p<0.01], accounted for by the slower responses for the hands-only (1118 ms) with respect to the hands-on-body (987 ms). In summary, changing the visual context in which a hand is presented (either attached to a human body or not) systematically affects response speed.

### Postural effects

**Figure 3:**
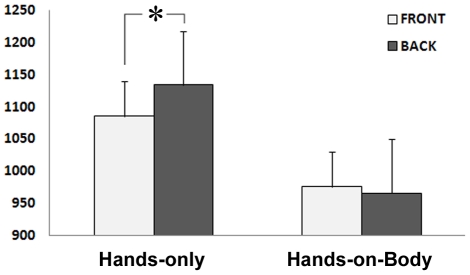
Hand posture affects mental rotation of hands-only but not hands-on-body stimuli. The posture-related difference of RTs varies between stimuli. Faster responses were found in the front postural condition with respect to the back postural condition for the mental rotation of hands-only stimuli. No differences due to postural changes were found in the mental rotation of hands-on-body stimuli. Error bars represent standard errors.

The significant stimulus by posture interaction [F(1,26) = 4.43, p<0.05] indicated a difference between the front and back posture for hands-only stimuli, but not for the hands-on-body stimuli (p<0.025; Fig. 3). In particular, when participants judged the laterality of hands-only keeping their own hands on the knees (“front” postural condition), performance was faster (1095 ms) with respect to when they judged hands-only keeping their own hands behind the back (“back” postural condition; 1141 ms). For the hands-on-body stimuli the difference between the front and back postural conditions was not significant (993 ms and 981 ms, respectively; p = 0.55).

### Other effects

We also found the frequently observed significant main effects of laterality [F(1,26) = 8.27, p<0.01], and orientation [F(3,78) = 30.76, p<0.01]. The main effect of laterality was accounted for by the faster performance for right (1035 ms) compared to left (1070 ms) stimuli. The main effect of orientation was accounted for by slowest responses for the stimuli oriented at 180° (1193 ms) with respect to all the other orientations (976 ms, 1017 ms, and 1025 ms for 0°, 90°, and 270°, respectively; all p<0.01). The 2-way laterality by orientation interaction [F(3,78) = 5.01, p<0.01] showed that the difference between right and left stimuli was significant only for stimuli oriented at 0° (950 ms and 1000 ms, respectively; p<0.03), and at 270° (979 ms and 1071 ms, respectively; p<0.01). The 2-way view by orientation interaction [F(3,78) = 16.56, p<0.01] showed that the difference between dorsum and palm view was significant for stimuli oriented at 0° (944 ms and 1007 ms, respectively; p<0.01), and at 180° (1239 ms and 1146 ms, respectively; p<0.01). The 4-way interaction between stimulus, laterality, view, and orientation [F(3,78) = 5.08, p<0.01] indicated that the modulation of the RTs according to the typical mental rotation function is preserved for both right and left hands-only stimuli, and is more pronounced for dorsum than palm view (especially for right-lateralized stimuli). Conversely RTs for both right- and left-lateralized hands-on-body stimuli were not modulated as a function of orientation (no difference between dorsum and palm view), and no differences were found between left- and right-lateralized stimuli. This shows that the typical mental rotation profile (non-monotonic increase of RTs as a function of stimuli's orientation) is preserved for hands-only but not for the hands-on-body stimuli. The absence of the orientation effect on mental rotation of hands-on-body stimuli is in line with previous studies [18], [19], [23], [31], and is probably due to the greater flexibility from physical laws reflected in the smaller dependency on biomechanical plausibility of the mental spatial reasoning performed on hands-on-body stimuli with respect to hands-only [21], [47].

## Discussion

In this study we demonstrate a clear dissociation between mental processes visual related to visual stimuli with different body context. In addition we show a differential and selective effect of body posture on such processes.

### Imagery strategy

The direct comparison between hands-only and hands-on-body performed in the present study provides insights into the differential influence of biomechanical constraints on two different strategy-dependent and stimuli-related cognitive processes. Classically during mental rotation of body parts, to more uncommon stimulus views correspond less orientation-dependent RTs [11]. The present study shows that this interdependence between stimulus orientation and view is absent for the mental rotation of hands-on-body while it is present for the hands-only stimuli. We controlled the visual aspects of the stimuli by using a target hand with the same physical features (e.g. posture, gender, age), view, and orientation in both the hands-only and the hands-on-body stimuli. While both tasks required judging hand laterality, the target hand was presented alone in the hands-only stimuli but it was attached to a body in the hands-on-body stimuli. Based on this we suggest that the differences in the dependency on view and orientation of the mental rotation of hands-only stimuli versus hands-on-body stimuli are due to context-related features of the stimuli. That is, by changing the bodily context in which a hand is presented (attached to a body versus alone) its mental processing will depend on differential cognitive mechanisms related to particular imagery strategies [9], [21], [48].

Which mechanisms are involved in the mental rotation of these two types of stimuli? The view-dependent disruption of the typical orientation-related RTs profile for mental rotation of hands-only has been consistently reported [review in 10]. This modulation of RTs has been related to the increase of biomechanical difficulty when simulating the hand rotation in order to match the progressively more difficult stimulus view [5]. In addition behavioural difference have been reported for mental imagery of anatomically possible versus impossible movements [7], [42]. Thus, in the mental rotation of hands-only the joint effect of stimulus view and orientation supports the fact that biomechanical joint constraints have an important role also for cognitive processing, such as the simulation of an action. The present study extends previous results by showing that, within the same population, RTs of the hands-on-body mental rotation are neither modulated nor differentially affected by stimulus orientation and view.

During mental rotation of hands-only, people generally imagine the outcomes of a simulated action as if they were actively performing that action [49]. However different imagery strategies can be elicited by different stimuli, and people can switch between them [1]. In the case of “perspective” transformations the relationship between the coordinate frames of objects and the environment remains fixed, and the self is used as a reference frame to determine the localization of e.g. an object. In the case of “effector-based” transformations (e.g. hand-centered) there is an update of the frames of reference with respect to the environment and the object [37]. We interpret the lack of the orientation-view interdependence of the mental rotation of hands-on-body, and its presence for mental rotation of hands-only, as evidence that two distinct stimulus-related mechanisms are recruited: “perspective” transformations are used for the hands-on-body stimuli; “hand-centered” transformations are used for the hands-only stimuli.

The increase of RTs due to biomechanical difficulty when mentally rotating hands-only in order to match the stimulus view, suggests that hand-centered transformations share at least some properties with manual actions [5], [12], [17]. In addition the activation of motor and premotor brain regions when performing mental rotation of hands [e.g. 5], [50], further suggests that the mental rotation of hands-only stimuli is based on sensorimotor mechanisms [43], [51], [52] and that it relies on kinesthetic and somatosensory representations [37], [47]. In contrast to hand-centered transformations, perspective transformations are not sensitive to biomechanical constraints [53], [54], are more physically flexible [47], are less dependent of anatomical plausibility [55], and are generally more complex [56]. In addition neuroimaging studies using hands-on-body stimuli showed the activation of temporal and parietal brain regions [e.g. 23], [57]. This suggests that perspective transformations are more based on visuo-spatial mechanisms. Interestingly, by adding a stereotyped “head” to the 3D objects classically used by Shepard and Metzler [58], RTs become less dependent on the stimulus orientation [59]. Based on the results of the present study it is possible to conclude that mental rotation of hands-on-body elicits a perspective transformation and is preferentially based on visuo-spatial mechanisms, while mental rotation of hands-only elicits hand-based transformations that rely mostly on sensorimotor mechanisms. Being less dependent on motor processes, perspective transformations (with hands-on-body stimuli) are not sensitive to changes in proprioceptive inputs such as postural manipulations, and to biomechanical joint constraints [53], [54].

How are hands-on-body stimuli mentally manipulated? Previous studies showed that the RTs profile for the laterality judgment of hands-on-body is modulated by the side (front versus back) of the human figure that is shown to the participants. Back-facing figures elicit processes similar to effector-based transformations, with the linear increase of RTs as a function of the stimulus orientation [e.g. 25]. This suggests that for correct task performance, it is sufficient to rotate only the plane of the sagittal body axis. Conversely front-facing figures are different in that the distribution of RTs is consistently reported as flat or only slightly dependent of the stimulus orientation [18], [22], [31]. This suggests that, in addition to the rotation of the sagittal body axis, a supplemental rotation of the longitudinal body axis is required, resulting in longer RTs [30].

It might be argued that the bodily context is not the only difference between the hands-only and hands-on-body stimuli we used. We cannot exclude the possibility that other visual features, such as identity and size of the target hand, might have had a role in the stimuli-related differences we observe. Yet, identity of a body part has a task-specific key role in different cognitive processes [e.g. 60]. However identity-related effects have not been reported in previous mental rotation studies that compared hands-only and hands-on-body [e.g. 18], or examined only mental rotation of hands-only [61], and as such it is unlikely that the target hands' identity might have a role in the bodily context effects we report. Furthermore, the size of the target hand has not been considered crucial by several studies that compared the mental rotation of hands-only, hands-on-body, and objects [30], [31], [34], [36]. Those studies (as did ours) controlled the overall size of the stimuli, and not the size of specific stimulus parts. Finally we cannot exclude the possibility that the presence/absence of two hands in the hands-on-body and hands-only stimuli respectively, might have an influence on mental rotation. In particular in the hands-on-body stimuli two hands were present, while in the hands-only stimuli only one hand was presented. However in both stimuli participants were requested to judge and focus on the laterality of only one hand. Nevertheless future work should investigate in particular the role of hand identity, size, and numerosity in the mental rotation of hands-on-body and hands-only stimuli.

### Body posture

As suggested above mental rotation of hands-only and hands-on-body differentially rely on kinesthetic/somatosensory and visuo-spatial representations, respectively. This difference is further supported by the presence/absence of influence of postural changes on their mental rotation. The present study shows that mental rotation of hands-on-body is not affected by the posture of participants' hands. Conversely hand posture affects the mental rotation of hands-only, as demonstrated by the performance decrease when participants held their hands behind the back. The influence of hand posture on mental rotation of hands-only has been consistently reported [9], [10], [29]. Up to now, no data were available on the role of kinesthetic input on mental transformation of hands-on-body stimuli. In the present study we used a within-subject design to show in a well-controlled manner that mental rotation of hands-on-body stimuli is less dependent on kinesthetic input relative to body posture, with respect to hands-only. Extending previous work, we show that the same posture manipulation does not interfere with mental rotation of hands-on-body. This suggests that anatomical joint constraints differently influence mental rotation of hands-on-body and hands-only and further indicates that these mental transformations rely on two different stimulus-related cognitive processes that are or are not sensitive to proprioceptive manipulations. The consistently reported role of postural information in the mental rotation of body parts indicates that kinesthetic representations are activated to some extent when dealing with mental images of body parts [16]. This also suggests that participants mentally simulate the movement of their own body in order to match the position of the presented stimulus, underlining the importance of kinesthetic information [5], [7], [10], [11], [29], [62]. The available data on the influence of motor and proprioceptive inputs on mental imagery of hands-on-body is controversial [30], [31], [47] and such mental transformations may rely rather on vestibular mechanisms [63]. The already mentioned possible dissociation between kinesthetic and visuo-spatial dominant mechanisms for mental rotation of hands-only and hands-on-body respectively, is further supported by clinical and experimental studies. In particular, studies that employed stimuli depicting human body-parts demonstrate stronger left parietal activations [64], [65], whereas studies using whole-body stimuli revealed more bilateral [23], [24] or right parietal and/or temporo-parietal activations [66]. Yet, neuropsychological findings suggest that the left hemisphere might be dominant for the processing of body-parts [16], [67], while own body illusions and deficits in corporeal awareness have been linked primarily to the right hemisphere [66], [68]. Accordingly, neuropsychological evidence showed that lesions in left fronto-temporal-parietal cortex determine a selective impairment in mentally rotation of hands [69], while lesions in right fronto-temporal-parietal cortex lead to selective impairment in whole-bodies mental transformations [36].

### Conclusions

The novelty and the theoretical advances of the present study can be summarized in four main points. First, this is the first time that the behavioural correlates of mental imagery of hands-only and hands-on-body are directly compared on a within-subject basis, providing strong evidence for the previously only hypothesized existence of different stimuli-related mechanisms. Our data show that visuo-spatial mechanisms are recruited during mental rotation of hands-on-body stimuli, while imagery of hand-only is preferentially based kinesthetic mechanisms. Second, we manipulated both stimulus orientation and view (dorsum and palm), showing that biomechanical constraints have an effect on mental imagery of hands-only, but not on hands-on-body. Third, we demonstrated that the view-dependent disruption of the typical orientation-related RTs profile is present for the hands-only but not for the hands-on-body. These view-related findings support that mental imagery of hands-only elicits effector-based kinesthetic mechanisms related to action simulation, while imagery of hand-on-body is based on perspective visuo-spatial transformations. Fourth, to our knowledge this is the first time that posture is manipulated during mental imagery of hands-on-body. Adopting this experimental protocol we support the involvement of visuo-spatial mechanisms, showing that proprioceptive information brought by posture does not affect mental spatial transformation of hands-on-body stimuli
